# Professional competences to promote healthy ageing across the lifespan: a scoping review

**DOI:** 10.1007/s10433-023-00794-7

**Published:** 2023-11-24

**Authors:** Elena Carrillo-Alvarez, Míriam Rodríguez-Monforte, Carles Fernández-Jané, Mireia Solà-Madurell, Mariusz Kozakiewicz, Mariola Głowacka, Mariel Leclère, Endrit Nimani, Adnan Hoxha, Armi Hirvonen, Sari Järvinen, Miriam van der Velde, Meike van Scherpenseel, António Alves Lopes, Hugo Santos, Isabel Guimarães, Marietta Handgraaf, Christian Grüneberg

**Affiliations:** 1 Global Research on Wellbeing (GRoW), Facultat de Ciències de la Salut Blanquerna, Universitat Ramón Llull, Barcelona, Spain; 2 Rydygier Collegium Medicum in Bydgoszcz, Nicolaus Copernicus University, Toruń, Poland; 3 Heimerer College, Pristina, Kosovo; 4 JAMK University of Applied Sciences, Jyväskylä, Finland; 5 HU University of Applied Sciences Utrecht, Utrecht, Netherlands; 6 Alcoitão School of Health Sciences, Alcabideche, Portugal; 7 HS Bochum University of Applied Sciences, Bochum, Germany

**Keywords:** Healthy ageing, Competences, Professional training, Scoping review

## Abstract

As societies age, the development of resources and strategies that foster healthy ageing from the beginning of life become increasingly important. Social and healthcare professionals are key agents in this process; therefore, their training needs to be in agreement with societal needs. We performed a scoping review on professional competences for social and health workers to adequately promote healthy ageing throughout life, using the framework described by Arksey and O’Malley and the Joanna Briggs Institute Guidelines. A stakeholder consultation was held in each of the participating countries, in which 79 experts took part. Results show that current literature has been excessively focused on the older age and that more attention on how to work with younger population groups is needed. Likewise, not all disciplines have equally reflected on their role before this challenge and interprofessional approaches, despite showing promise, have not been sufficiently described. Based on our results, health and social professionals working to promote healthy ageing across the lifespan will need sound competences regarding person-centred communication, professional communication, technology applications, physiological and pathophysiological aspects of ageing, social and environmental aspects, cultural diversity, programs and policies, ethics, general and basic skills, context and self-management-related skills, health promotion and disease prevention skills, educational and research skills, leadership skills, technological skills and clinical reasoning. Further research should contribute to establishing which competences are more relevant to each discipline and at what level they should be taught, as well as how they can be best implemented to effectively transform health and social care systems.

## Introduction

The World Health Organization (WHO) defines healthy ageing as the process of developing and maintaining the functional ability that enables well-being to an older age (WHO 2020b). Demographic data from European regions show a generalised trend towards aged societies. As life expectancy continues to increase and birth rates lower, not only will the composition of European countries change, but their health landscape also will (Beard et al. 2016; European Commission 2021). In 2019, life expectancy at birth was 78.5 years for men and 84 years for women. However, more than 20% of those years are lived in ill health due to chronic conditions caused by noncommunicable diseases (NCDs), which compromise functional ability and well-being—key components of healthy ageing (WHO 2018). In this way, not only people's lives, but also societal structures are being affected. The strain on healthcare services is rising steeply, as are costs, to the point that if nothing is changed, they will reach completely unsustainable levels in most OECD countries (OECD 2015). Along the same lines, the European Commission’s Green Paper on Ageing establishes the need to find new solutions to support people and to adapt and increase funding for health and care systems to cope with the higher age-related public spending (European Commission 2021).

Commonly described NCDs are often accompanied by growing comorbidities and societal problems, such as functional decline, frailty syndrome, loneliness, dementia and depression, especially among the elderly (Bevilacqua et al. 2022; Delerue Matos et al. 2021; Livingston et al. 2020)—and a widening of social and health inequalities within and among countries (Bono and Matranga 2019; Peñalvo et al. 2021; Sadana et al. 2016). In fact, being disease-free is not a requirement to enjoy healthy ageing, since the definitory characteristic of healthy ageing is functional ability, understood as 'the person’s ability to meet their basic needs; learn, grow and make decisions; be mobile; build and maintain relationships; and contribute to society' (WHO 2020a), which depends on both the intrinsic capacities of individuals and their environment and can coexist with illness if they are well regulated. In any case, research shows that morbidity increases the probability of reporting functional limitations, both of which lead to poorer self-rated health. Therefore, promoting healthy ageing involves both enhancing functional ability and preventing disease.

Currently, the majority of efforts to prevent and treat the impact of ageing are placed in the last stages of life (United Nations 2018, 2019, 2020). However, it will not significantly change the current struggles of health and social systems globally, since the process of ageing starts from early childhood; therefore, resources should be invested to focus on its importance from the beginning of life (WHO 2021).

It has been stated that health and social care professionals are not adequately trained to support the rising ageing of European populations, particularly in regard to the implementation of a life-course approach to healthy ageing. Strengthening the investments in health promotion and disease prevention is key to delaying the onset and reducing the burden of NCDs in Europe (Barnfield and Savolainen 2019). Although there is evidence that health promotion, disease prevention and lifestyle medicine have been growing in the curricula of most European health professions (Santa-María Morales et al. 2009), a recent European report stated that described competences are not ranked in order of importance and it is often not explained how the competences should be applied in practice (ECORYS 2020). Therefore, there is a need to improve the description of many competency profiles in this field.

The SIENHA project (Strategic, Innovative, Educational Network for Healthy Ageing 2020-1-ES01-KA203-083121) is an EU-funded initiative that aims to tackle this situation by (1) transforming healthcare and social systems through the education of future managers and employees in order to integrate a clear vision of sustainably fostering healthy ageing throughout life; (2) equipping future health and social care workers with state-of-the-art skills and competences by developing a qualitative educational framework; (3) supporting transdisciplinary innovation and creative partnerships that strengthen the role of higher education at a regional level; and (4) ensuring that education and research are mutually reinforcing, thus incentivising and rewarding effective teaching practices.

To do this, the project establishes an educational network with the aim of building a knowledge platform centred on healthy ageing education. SIENHA will develop education content and methods in healthy ageing at undergraduate, postgraduate, vocational and non-formal levels. The framework will include the competences that need to be achieved at each level to ensure the proper management of healthy ageing across the lifespan. It will also comprise the contents, as well as pedagogical and research strategies that better contribute to achieving those competences. The partners that conform the SIENHA Project belong to seven countries with different cultural and educational realities: Finland, Germany, Kosovo, Poland, Portugal, The Netherlands and Spain, therefore making this competence framework relevant across Europe by capturing cultural and educational particularities of these contexts.

This paper reports the results of a Scoping Review with the aim of identifying the competences that have been described for health and social professionals to promote healthy ageing across the lifespan.

## Methods

This review is reported in accordance with the reporting guidance provided in the Preferred Reporting Items for Systematic Reviews and Meta-Analyses (PRISMA) Extension for Scoping Review Checklist" (Tricco et al. 2018). The protocol of this review can be accessed at the website of the project (www.sienha.eu).

This study follows the methodological framework described by Arksey and O’Malley (2005) and the Joanna Briggs Institute guidelines (Pollock et al. 2021). The framework involves a six-stage phase: (1) identifying the research question; (2) identifying relevant studies; (3) selecting studies; (4) charting the data; (5) collating, summarising and reporting the results; and (6) consulting with key stakeholders. In the following pages, we describe how these phases have been conducted in the context of Intellectual Output 1 of the SIENHA project.

### Step 1: identifying the research question

Following Arksey and O’Malley’s recommendation to avoid dealing with a “high focused research question”, the scoping review was conducted based on the following research question: *What competences have been described for social and health professionals to adequately promote healthy ageing over the life course?* In this way, we aim to identify competencies that should exist in the curricula of the health and social professionals that deal with healthy ageing.

### Step 2: identifying relevant studies

In order to achieve the answer to the central research question, we adopted a strategy that involved searching for documentation via different sources: scientific literature and grey literature.

After consultation with an academic librarian, a search strategy was designed for Medline/PubMed and adapted to the other databases. Specific search strategies used for each database can be consulted in annex I. The construction of the search strategy and equation as well as the establishment of inclusion and exclusion criteria were achieved through an iterative process of refinement to maximise the comprehensiveness and relevance of the results (Higgins et al. 2019). For instance, after a first screening phase, we realised that most of the identified papers included a description of competencies linked to health promotion but did not specifically address healthy ageing. While we recognise that health promotion is, in itself, a way of promoting healthy ageing across the lifespan, we aimed to identify the professional competences purposely described to foster healthy ageing. Consequently, a further inclusion criterion was added: to only include documents that specifically include the term “healthy ageing”. Likewise, in our preliminary search design, we included the terms “knowledge”, “skills”, “capabilities” and “abilities” to try to cover all aspects related to competences. However, this strategy resulted in an enormous number of results, most of them not related to our research question. Consequently, we finally decided to develop a more specific strategy gathering all of the previous terms in the main concept, which is *"*competence".

A scientific literature search was conducted in a centralised manner by the leading team of intellectual output1 of the SIENHA Project, through the following health and social science bibliographic databases: PubMed, Cochrane Library, Scopus, CINAHL, Web of Knowledge, PsychInfo, ERIC, SportDiscus. Grey literature was identified by all partners of the SIENHA Project. Each partner looked for grey literature published in their national languages through the translation of the research question to the respective languages: Albanian, Catalan, Dutch, German, Finnish, Polish, Portuguese and Spanish. Grey literature in English was also searched for by all teams. The term ‘Grey Literature’ includes the following potential documents: (1) Reports by Governments and NGOs; (2) Dissertations/Theses; (3) Newsletters and Press articles; and (4) Conference presentations. As there is not yet any gold standard for searching grey literature, the search was carried out via search engines such as Google, Yahoo, Google Scholar and Open Gray, specific websites such as UE, each country's government, non-governmental sources, thesis repositories, etc. and University library websites that offer a comprehensive list of grey literature databases.

The eligibility criteria considered the following information:

- *Type of Publication* all kinds of original studies, review articles, guidelines, etc.
- *Outcome*
  - Competences described as being necessary for optimal professional performance and/or taught at the levels of vocational training, bachelor, or masters’ degrees.
  - Competences required to promote healthy ageing at any point of the life course.
  - Competences referring to social or health professionals that work with people: such as psychologists, social workers, teachers, nurses, physiotherapists, dietitians, physicians, dentists, pharmacists, physical educators and sport scientists.
- *Timeframe* January 2000 to March 2021
- *Language* sources written in the participants’ native languages and English
- *Context* in order to identify competences that are relevant for European countries, we included documents from culturally similar contexts: EU countries, the United Kingdom, the United States, Canada or Australia.

All articles describing competences referring to patients, competences to promote health among individuals with health conditions other than the five most prevalent NCDs in Europe: chronic respiratory diseases, cardiovascular diseases, diabetes, cancer and mental disorders, or study protocols were excluded. Studies describing the current competences that professionals had or perceived to have were also discarded.

### Step 3: selecting studies

Covidence screening and data extraction tool was used for this process. A two-step screening process was used to identify the relevance of documents in the search. Firstly, the titles and abstracts of the studies or other types of documents such as reports, or guidelines identified in the search were reviewed independently in pairs considering the eligibility criteria. Secondly, all potentially relevant documents were obtained in full text and also reviewed in pairs. Any discrepancies were resolved by consensus. If the consensus was not achieved, a third investigator was included in order to reach agreement.

### Step 4: charting the data

Data were abstracted by two independent reviewers and compared. A standardised form created by the research team in order to collect the data (Microsoft Excel Spreadsheets) was followed as well as the JBI Reviewers’ Manual 2015 for data charting. The grid prepared to extract these data can be found in annex II. The data extracted included the following information: authorship; year of publication; country where the study was developed; language; source (type of document); definition of healthy ageing; moment of the life course to promote healthy ageing; competences; educational level to which competences apply; professionals to which competences apply; and email of the corresponding author.

### Step 5: collating, summarising and reporting the results

The main findings were summarised using a narrative descriptive synthesis approach and grouped following the domains described in the definition of professional competence proposed by Epstein and Hundert (2002), according to which professional competence is “the habitual and judicious use of communication, knowledge, technical skills, clinical reasoning, emotions, values and reflection in daily practice for the benefit of the individual and community being served”. Although professional competences clearly arise from the integration of the former domains, we apply this differentiation to organise this scoping review in a clearer manner.

Based on the type of individual sources of evidence found, which were mostly from grey literature databases and following the framework of Arksey and O’Malley, no quality assessment was conducted.

### Step 6: consulting with key stakeholders

We designed and adapted a strategy that brought together different purposeful samples of stakeholder profiles in order to present and enrich the preliminary results of the scoping review. Following this approach, each partner of the SIENHA consortium contacted different stakeholders from their country and invited them to participate in an online meeting. The strategy for the consultation with stakeholders took into account the recommendations described by Arksey and O’Malley, Levac, Colquhoun and the Joanna Briggs Institute guidelines.

Participants were verbally sought for consent to share their views, a procedure clearly outlined in the invitation letter that detailed the consultation's purpose and process. To uphold confidentiality, all collected information remained anonymous, shielding participants' identities. Utilising the Google Meet platform for online group meetings, the interactive sessions facilitated dynamic discussions and idea sharing. Participants were informed that their main ideas would be transcribed using standardised forms, ensuring transparency. These transcriptions not only captured the essence of discussions but also served as a valuable tool for synthesising and contrasting stakeholders' thoughts with existing literature, aligning seamlessly with the primary objective of the consultation.

Phases that included:

*Phase 1: Defining the purpose of the consultation*. In the context of our scoping review, the goal of the consultation was to disseminate our preliminary results as well as to contrast them with a group of stakeholders. Additionally, we aimed to identify unmet needs to promote healthy ageing across the lifespan in ageing populations and the competencies that should exist in the curricula of the health and social professionals that deal with healthy ageing.

*Phase 2: Definition of the profile of the stakeholders*. Following the objective to maximise the diversity and gather relevant input from different stakeholder, we sought to invite: (a) professionals of health or social disciplines such as psychologists, social workers, teachers, nurses, physiotherapists, dietitians, physicians, dentists, pharmacists, physical educators, sport scientists included in phases 1–5 (10–12 per partner); (b) citizens at different life stages: adolescent, young adult (25–40), middle-age adult (40–65), older adults (> 65), (1 of each group, total of 4 per partner) and, if possible, from different socioeconomic backgrounds; (c) citizens that influence decisions on the content of educational programs: academic vice-rectors and vice-deans, professional associations, national platforms for the education of different professionals, etc. (6–8 per partner); and d) representatives of public administrations dealing with healthy ageing: health, social and welfare departments at different levels (city, region, country…) (2–3 per partner).

*Phase 3: Defining a date and space for developing the consultation and inviting the stakeholders*. Each partner of the SIENHA consortium sent an invitation with at least three weeks’ notice to the stakeholders that met the characteristics of the previously described profiles. Due to the COVID situation, the meetings were held virtually. All countries' consultations took place between 15 May2021 and 10 June2021.

Phase *4: Undertaking the consultation*. The consultations lasted around 2–2:30 h and had three different parts. In the first one (45 min), the preliminary findings of the scientific and grey literature search were presented, emphasising the results of each country. A common template later translated to each country's national language was used for homogeneity purposes. The covered topics included: greetings, justification of the invited profiles and explanation of the activity procedure, overview and main aims of the SIENHA Project, definitions of healthy ageing (across the lifespan), notion of competence and results of the literature review on competences to promote healthy ageing. The second part of the consultation took part in small groups of 5–6 invitees where they shared their views and experiences linked to the purpose of the consultation. It lasted for 30 min and sought input on three preliminary questions: overall feedback on the previously presented results; concept of healthy ageing and related societal needs; and competencies that should exist in the curriculum of health and social care professionals involved in healthy ageing. We also asked the different stakeholders to focus on whether these competencies should be more specific to a particular professional profile, or should be addressed in a multidisciplinary manner. In the third part of the meeting, stakeholders shared their group conclusions (40 min) with the rest of the participants. At least two or three SIENHA team members of each country attended the consultation session. Notes were taken by a member of the team in each small group and through the plenary part of the meeting.

Phase *5:* Summarising the results. After the consultation session, each national team met and shared their notes, and reached consensus on the final report. The final report was sent by each partner to the project coordinating team containing a summary of the stakeholder meeting and considering the following information: number and profile of the participants; overall impression of the stakeholders on the results of the literature review; specific feedback on the adequacy, pertinence, and relevance of the identified competences—including the proposal of new ones; and anything that the group wanted to highlight. Last, the coordinating team merged the feedback of the stakeholders with the competences identified in the literature review following the same conceptual organisation.

## Results

### Literature review

**Fig. 1:**
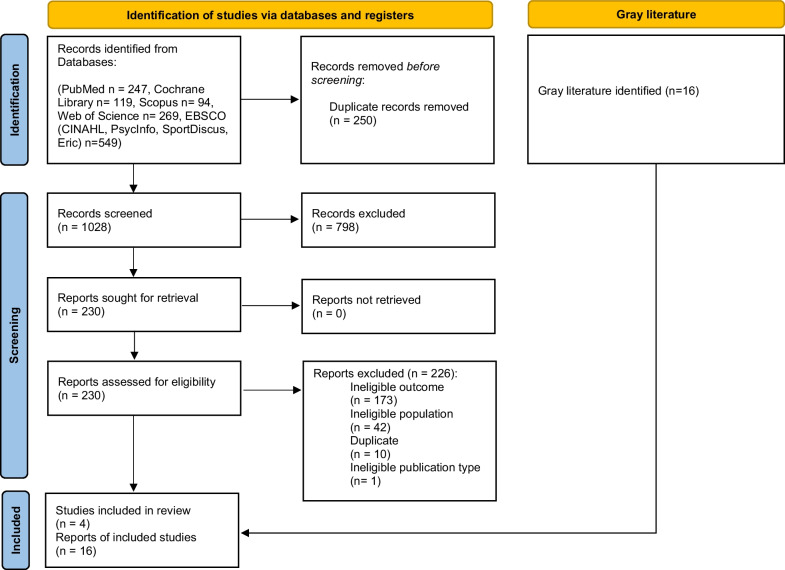
PRISMA flow diagram

Regarding the scientific literature, we obtained 1278 records in our initial search. After removing 250 duplicates, we screened 1028 records by title and abstract, removing 798 irrelevant records and leaving 230 full-text reports. Finally, and after excluding 226 reports, we included four studies. Regarding the grey literature, we included 16 documents. Therefore, overall, we identified 20 documents. The selection process is summarised in Fig. 1.

The general characteristics of the reviewed studies/documents, including the competences described for social and health professionals to adequately promote healthy ageing, are listed in Table 1.

**Table 1:** General characteristics of the reviewed studies and documents

Authors, year	Stage of life for promoting healthy ageing	Competences needed by health and social professionals to promote healthy ageing	Educational level to which competences apply	Professional to which competences apply
Australian College of Nursing (2019)	Older adults	Promoting and demonstrating a positive view of ageing including respect and empathy for the older person Effective communication including verbal and documentation skills and assessing the barriers older adults encounter receiving, understanding, and giving information Reflective practice skills Knowledge of the aged care system and available aged care services Maximising health outcomes through knowledge of the ageing process Recognition of the complex interaction of acute and chronic comorbid physical and mental conditions and associated treatments common to older adults Skills to provide optimal pain management and palliative care Enabling older adults access to technology Incorporate professional attitudes, values, and expectations about physical and mental ageing in the provision of person-centred care for older adults and their families Commitment to comprehensive assessment of the person and individualised identification of client need Assess the living environment and community resources Commitment to client empowerment and choices within legal and ethical frameworks Implement strategies and use online guidelines to prevent and/or identify and manage geriatric syndromes Recognise and respect the variations of care, the increased complexity, and the increased use of healthcare resources in caring for older adults Provide person-centred care with consideration for mental and physical health and well-being of informal and formal caregivers of older adults Implement and monitor strategies to prevent risk and promote quality and safety in the nursing care of older adults with physical and cognitive needs Utilise resources/programmes to promote functional, physical, spiritual and mental wellness in older adults	NA	Nurses
Beenen (2018)	NA	Professional identity Critical conception of knowledge and paradigms Creativity in design Leadership in innovation processes Setting up innovation project Practice-based research	Postgraduate (Master)	NA
Bernett (2017)	Older adults	To seek education regarding biological, psychological, cultural, and social concerns related to ageing Be aware of the necessary adaptations and changes in relationship dynamics, and the impact that these changes can have on these clients To understand the impact of diversity on the ageing process and how these factors can influence the experience of health and mental health issues for older adults Be aware of these differences and be willing to understand the unique experiences of each individual when treating them Be familiar with information about biological and health-related aspects of ageing Be able to distinguish normative from non-normative aspects of ageing and be able to provide coping strategies Be knowledgeable about common health concerns, sexual functioning, and information about commonly prescribed medications and possible interactions Be familiar with cognitive changes associated with the ageing process To understand the functional capacity of older clients in their environments and how changes in functional capacity can impact their lives Be knowledgeable about and address the difficulties older adults may experience in their relationships, physical environments, and psychological wellbeing when functional capacity is altered by ageing Be knowledgeable about the current culturally relevant methods of assessment and instruments of assessment for older adults and ensure that these measures used are appropriate for this specific population To have the ability to properly accommodate assessment contexts based on the needs of clients, including physical, sensory, or psychological limitations Be knowledgeable regarding research on treatment, therapeutic interventions, and the efficacy of such interventions for practice with older adults Be familiar with applying culturally sensitive interventions in practice and possess the necessary skill set to modify treatments based on the needs To work collaboratively with other disciplines (and educate ourselves about each discipline’s role in the evaluation and treatment of older adults) and making referrals to other disciplines To increase the skills, knowledge, and understanding related to working with older adults through training, supervision, consultation, and continuing education	N/A	Mental health Professionals
Degenaar (2018)	Life course	To strengthen self-management and resilience of patients To contribute to prevention and health literacy of citizens To focus on functioning and the ability to adapt and self-manage To use interdisciplinary innovative methods in health improvement like health technology	N/A	Nurse
Dijkman (2019)	Older adults	Advocate for health with, and on behalf, of older persons and their families, communities, and organisations Access and share information or resources with older persons, their families, and their caregivers, regarding the social map, healthcare benefits, social support and public programs	Bachelor level	Health and social care professionals
EUHPP Thematic Network (2021)	Life course	Adaptability to different settings and models of care Person-centric communication skills and empathy towards the patients Digital skills Basic health data analysis skills Management skills Interdisciplinary teamwork skills Administrative skills	N/A	N/A
EuroHealthNet (2020)	N/A	Skills in communication training Skills in interprofessional training Getting to know the neighbourhood Structured approaches for complex patients Bridges to Health Model	Bachelor level and postgraduate	General practitioners
Faculty of Medicine of the University of Porto	N/A	To deepen knowledge about the political, biopsychosocial, cultural and bioethical factors associated with normative to pathological ageing Develop skills within the scope of promoting active and healthy ageing Develop skills within the scope of rehabilitation of elderly individuals Develop knowledge in the context of the design, promotion and evaluation of interventions To deepen knowledge in the context of support to formal and informal caregivers involved in the relational spheres Develop an integrated and multidisciplinary approach adjusted to the needs of the healthy elderly, the sick and terminally ill elderly	Postgraduate (specialisation course)	Graduates or holders of a Master's Degree in Health Sciences and Humanities and Social Sciences, Sports Sciences and Educational Sciences
Frank (2014)	Older adults	Understand the trajectories of improvement and/or decrement in individual functioning Understand state and national ageing policy and programs Establish rapport and sustain effective working relationships with a wide range of older adults, their families, and caregivers Define/describe the bio/psycho/social concepts and theories used to study ageing Identify how an older person is affected by the person–environment interactions Knowledge of general ethical principles and how they relate to professional practice in gerontology Understand common threats to loss of independence: falls, medication management, and lifestyle Understand the role of social service, community recreation, and health service providers in their involvement with older persons Maintain currency in research findings of evidence-based disease management programs, including frameworks, theories, and models Facilitate elders’ and families’ adaptive capacity related to disease and geriatric syndrome management Understand the requisite practice skills appropriate to areas of gerontological practice Understand the importance of program review and evaluation for program effectiveness Understand issues of diversity among older adults and their families	Bachelor level and postgraduate	Ageing services personnel
Hiltsje (2015)	Older adults	To be able to describe determinants of healthy ageing from an integrative biopsychosocial perspective	Bachelor level	Doctor, physician
Grupo de Serviço Nacional de Saúde. Trabalho Interministerial (2017)	Older adults	Specific skills in caring for the elderly Skills to develop and execute Individual Care Plans, based on the promotion of health and functional abilities, and that take into account the needs private individuals of women and men Skills to intervene in the optimisation of functional capacity Competences for an integrated response to the problems of the elderly (multidisciplinary teams) Skills in promoting healthy lifestyles and health surveillance Management of comorbidity processes Education and training throughout the life cycle Creation of environments that enhance integration and participation Creation of physical environments that guarantee security Identification, signalling and support in situations of vulnerability Measurement, monitoring, and investigation	N/A	Health professionals: doctors, physiotherapists, speech therapists, occupational therapists, nurses, rehabilitation nurses, Social Work Professionals; Pharmacists; Dentists, Nutritionists; Psychologists
NetwerkZON (2020)	HA relates to the entire life course and begins as early as the period before conception, when parents pass on their genes, thus with possible risks and opportunities for a healthy life course	Professionality in permanent development Collaborating within teams and across their own (discipline/organisation) boundaries (among professionals and with community residents) Knowledge of the client's various environmental factors: such as social networks, society, housing situation, work situation, living conditions and money flows Organising and planning Taking initiative and direction, but also setting boundaries Accountability and reflecting	Vocational level	Health care and welfare professional
Pinto (2020)	N/A	To know ageing theories To have the ability to interfere in the adherence to treatment plans To promote actions to prevent falls and their consequences To prevent frailty, physical and cognitive decline To optimise media among health professionals and integrated clinical services To promote active life during ageing To improve and maintain the self-management skills To be able to advise healthy eating habits To promote good practice in the treatment of elderly patients	Non-degree lecture course	Elderly caregivers, with preference to health professionals
Poulos et al. (2021)	Older adults	Describes the social determinants of health, key risk factors, and individual behaviours that influence health over the life course Applies evidence-based approaches to screening, and disease and injury prevention for older people Advocates to older people (and their informal caregivers) interventions and behaviours that promote health and wellbeing, while respecting older peoples’ autonomy Provides appropriate options, information, resources, and education to empower older people (and their informal caregivers) to actively participate in maximising their functional ability and achieving their chosen goals Identifies and effectively responds to the health literacy, health education, and health promotion needs of older people (and their informal caregivers) Raises awareness in the wider community about healthy ageing and the needs of older people	Postgraduate	Health and aged care workers
Rodger (2017)	Older adults	To create an awareness of the potential for falls in older adults and reduce the risk of falls in an ageing population To identify the medium to present the education To identify the need to engage with an educational technologist to develop the online education material To design clear and locally tailored resources to address stakeholder problem needs	Postgraduate (Master)	Advanced Nurse Practitioners
Romero (2020)	Ageing from retirement	To stimulate socially and culturally, entailing the promotion from motivation to social participation and the dynamisation To design, develop, manage, and evaluate plans, programs, projects and activities To encourage and improve active ageing, generating resources educational and social issues To generate educational contexts that promote learning in this vital stage, advocating for lifelong education To advise and accompany the elderly in processes of socio-educational development, favouring its growth personal, autonomy and empowerment To encourage social education (socially educate) through the socio-educational intervention To foster relationships, including those of character intergenerational, with a tendency to avoid people’s loneliness great, working on an emotional and socio-affective level, thereby improving communication and repercussions on their mood Starting from the knowledge of the context to carry out interventions contextualised To coordinate, manage and organise resources, activities, people and institutions To detect deficiencies and socio-educational needs to be able to cover them and / or solve them To encourage new possibilities through the provision of resources and information and the creation of spaces for participation To encourage quality and creative leisure providing a socio-educational component	N/A	Social worker (Social educator)
Wiekens (2019)	Life course	Systematic approach Evidence-based approach Person-oriented approach Network-centred approach	Bachelor level	Applied Psychology, Applied Gerontology, Social Work, Nursing, Physiotherapy, Paramedics, physicians and lifestyle coaches
WHO (2016a)	Life course (people in the second half of their lives)	To have gerontological and geriatric skills Be able to share information using information and communication technologies To provide self-management support To work in multidisciplinary teams	N/A	N/A
WHO (2016b)	N/A	Be able to identify neglect or abuse Be able to perform basic screening for physical and mental capacities as well as nutritional status and oral health Be able to manage common health conditions faced by older populations	N/A	Health-care professionals, with emphasis on medical doctors
World Health Organisation, and ASPHER (2020)	Life course	To have competencies on science and practice: epidemiology of communicable and noncommunicable diseases; demography; biostatistics; qualitative and quantitative research methods; assessment, analysis and evaluation; evidence-based research; measurement, monitoring and reporting; health indicators; health systems; population health; health inequalities To have competencies on promoting health: education and promotion through social participation; health literacy at the community, organisation and individual levels; citizen empowerment; health needs assessment; screening and secondary prevention; evaluation of health promotion interventions and programmes To have competencies on content and context law, policy and ethics: international and European laws and regulations; European public health law; strategies and strategic approach (international, national and local levels); policy development and planning; programme and policy assessment and implementation; priority setting; ethics, ethical frameworks, ethical practice and decision-making To have competencies on one health and health security: human health; health protection; occupational health; food safety; animal health; cross-border health; international health; global risks and threats; preparedness and response; pandemics; environmental health; climate change To have competencies on leadership and systems thinking: vision, mission and strategy; individual task-team work; leading change and innovation; understanding and applying the theories of complex systems in practice; organisational learning and development; people development; emotional intelligence To have competencies on collaboration and partnerships: effective collaboration; building alliances and partnerships; networking and connecting; working with and building interdisciplinary and intersectoral networks; dealing with and managing stakeholders To have competencies on communication, culture and advocacy: effective written and verbal communication, including communication with the media; scientific communication; presentation, respect for diversity and inclusiveness; historical and cultural context; advocacy and diplomacy To have competencies on governance and resource management: human resource management; organisation, administration and governance of resources; financial planning; quality assurance; technical expertise and logistics; basics of health economics; economic evaluation and analysis To have competencies on professional development and reflective ethical practice: professional and reflective practice; continuing professional development; lifelong learning; values; ethical professional conduct To have competencies on organisational literacy and adaptability: use of technology; data management; entrepreneurship; fundraising; creativity, analysis and synthesis; digital health and social media; understanding of public health services and operations	N/A	Public Health professionals

In terms of the *life stage to which the competencies that professionals should develop apply*, almost half of the documents are focused on the older adult population (Australian College of Nursing (ACN) 2019; Barnett and Quenzel 2017; Dijkman et al. 2019; Frank et al. 2014; Grupo de Serviço Nacional de Saúde. Trabalho Interministerial 2017; Heemskerk and van Bodegom 2015; Martínez de Miguel López et al. 2020; Poulos et al. 2021; Rodger and Hussey 2017), whereas six documents describe competences to promote healthy ageing across the life course (Degenaar 2018; Health First Europe and EHMA 2021; Netwerk ZON 2020; WHO 2020a; Wiekens et al. 2019; WHO 2016b).

Regarding the *professionals to which the identified competences apply*, ten of the included documents describe several professionals from different disciplines (Dijkman et al. 2019; FMUP 2014; Frank et al. 2014; Grupo de Serviço Nacional de Saúde. Trabalho Interministerial 2017; Netwerk ZON 2020; Poulos et al. 2021; Universidade de Coimbra 2020; WHO 2020a; Wiekens et al. 2019; WHO 2016b). Moreover, seven studies describe competences for a specific profession including: nurses (Australian College of Nursing (ACN) 2019; Degenaar 2018; Rodger and Hussey 2017), social workers (Martínez de Miguel López et al. 2020), medical doctors (Heemskerk and van Bodegom 2015; WHO and ASPHER 2020) and mental health professionals (Barnett and Quenzel 2017). Three studies did not report the professionals to which the competences applied (Beenen et al. 2018; Health First Europe and EHMA 2021; WHO 2016a).

The *educational level to which competences apply* ranges from undergraduate to postgraduate training. One document refers to a non-degree lecture course (Universidade de Coimbra 2020), three of the documents refer to bachelor level (Dijkman et al. 2019; Heemskerk and van Bodegom 2015; Wiekens et al. 2019), four refer to postgraduate level (Beenen et al. 2018; FMUP 2014; Poulos et al. 2021; Rodger and Hussey 2017) and two include both bachelor and postgraduate training (Frank et al. 2014; WHO and ASPHER 2020). One of the documents attributes competences at a vocational level (Netwerk ZON 2020) and nine documents do not indicate the educational level (Australian College of Nursing (ACN) 2019; Barnett and Quenzel 2017; Degenaar 2018; Grupo de Serviço Nacional de Saúde. Trabalho Interministerial 2017; Health First Europe and EHMA 2021; Martínez de Miguel López et al. 2020; WHO 2016a, 2020a; WHO 2016b).

We identified a large variety of competences (n = 142), which were grouped following the domains described in the definition of professional competence proposed by Epstein and Hundert (2002): communication, knowledge, technical skills, clinical reasoning, attitudes and reflection. Across the included documents, different terminology is used to refer to the individuals to whom healthy ageing activities are addressed: patients, persons, individuals, users and clients. For simplicity purposes, we always refer to users.

In the domain of *Communication*, three thematic areas were identified: *communication with the users, families, caregivers and the community*; *professional communication*; and *technology*. Competences related to *communication with the users, families, caregivers and the community* included the ability to create rapport and sustain effective working relationships with a wide range of older adults, their families and caregivers and to show empathy towards users (for example by understanding the unique experiences of each individual, respecting their uniqueness and taking into account wishes, preferences and the possibilities to self-manage). In the *professional context*, communication competences included the development of integrated and multidisciplinary approaches, working collaboratively with other disciplines and organisations nationally and internationally and fostering open and honest professional relationships, making referrals to other disciplines, as necessary. With regard to *technology*, the need to adequately use communication and health technologies when sharing information was described.

In the domain of *Knowledge*, we identified seven thematic areas: *physiological and pathophysiological aspects of ageing*; *social and environmental aspects of ageing*; *cultural diversity*; *programs and policies*; and *ethics*. *Physiological and pathophysiological aspects of ageing* included having knowledge of the biological and health-related aspects of ageing and of the health concerns when functional capacity is altered by ageing, as well as being aware of concepts and theories to study ageing from a biopsychosocial approach. The area about the *social and environmental aspects of ageing* comprised having knowledge of the user's various environmental factors (i.e. social networks, society, housing situation, work situation, living conditions and money flows) and difficulties that can arise in these areas. The literature also highlighted competencies related to the importance of knowing the role of social service, community recreation and health providers for older people as well as the implications of health inequalities and inequities for active and healthy ageing with reference to social determinants of health. *Cultural diversity* was amongst the most referenced competences for healthy ageing, and it includes understandings of the impact of diversity in the ageing process and its influence on health and mental health issues in older adults, of issues of diversity in older adults and their families and of current culturally relevant methods and instruments of assessment for older adults, as well as research regarding these assessment methods. Within the domain of *programs and policies*, the competencies comprised being aware of the existing national and international policies and programs on ageing, as well as having knowledge of how to evaluate program effectiveness. Last, the literature referred to knowledge of the *ethical principles*, *law and politics* related to healthy and pathological ageing and to gerontological practice.

The domain of *Technical Skills* includes six thematic areas: *general and basic skills*; *context and self-management skills*; *health promotion and disease prevention skills*; *educational and research skills*; *leadership skills*; and *digital skills*. *General and basic skills* involve evidence-based skills for healthy ageing and in gerontological practice, the ability to properly accommodate assessments for healthy ageing based on users’ needs including physical, sensory, or psychological limitations and the ability to provide integrated management of common conditions and complex health care needs faced by older populations, performing according to patient safety principles, identifying neglect or abuse and engaging with people as individuals in a culturally safe and respectful way. The area of *context and self-management skills* includes the ability to identify how an older person is affected by person–environment interactions, to identify, signal, measure, monitor and provide support to those in vulnerable contexts, to facilitate elders’ and families’ adaptive capacity related to disease and geriatric syndrome management, to favour users’ personal growth, autonomy and empowerment, to access and share information with older people/patients, families, caregivers, to promote shared decision-making and care delivery between the person, nominated partners, family, friends and health professionals and to focus on functioning and the ability to adapt as to strengthen self-management and resilience of the patient. It also includes advising and accompanying the elderly in processes of socio-educational development as well as providing coping strategies for clients struggling with changes. With regard to *health promotion and disease prevention skills*, the reviewed literature refers to promoting health and well-being for people and their families, colleagues, the broader community and themselves in a way that addresses health inequality, promoting active and healthy ageing considering personal independence and social participation, designing and evaluating clear and locally tailored resources to address stakeholder problem needs, developing and promoting actions to senior population within the scope of health, education and psychosocial fields, encouraging social education (socially educate) through the socio-educational intervention, contributing to promote the health literacy of citizens and developing specific knowledge to support informal and formal caregivers involved with elderly people and creating an awareness of the potential for falls in older adults and reduce the risk of falls in an ageing population. *Educational and research skills* refer to generating educational contexts that promote learning in this vital stage, advocating for lifelong education, having basic health data analysis skills and identifying the medium to present education in order to reach a wide audience. *Leadership skills* include organising and planning, governance and resource management, setting up innovation projects, management skills, leadership in innovation processes, creativity in design, the generation of resources for educational and social issues that favour active ageing, and the stimulation of social participation for the overall population and for the older people in the community. Digital skills were referred to in general.

The domain of *Clinical Reasoning* includes understanding the unique experiences of each individual when treating them and distinguishing normative from non-normative aspects of ageing. The domain of *Attitudes* comprised integrating the existing professional identity with the one of ‘change agent’, focusing on the transition of healthy ageing, taking initiative and direction and also setting boundaries, adhering to obligations about privacy and confidentiality, being able to adapt to different settings and models of care, being aware of the necessary adaptations and changes in relationship dynamics and the impact that these changes can have on these clients to provide them with competent services and combating ageism and seeking lifelong education to meet the needs to ageing. Last, in the *Reflection* domain, accountability and a reflective ethical practice were included.

### Stakeholder consultation

In total, 79 individuals participated in the stakeholder consultation, whose profile is shown in Table 2. Most of the participants had a professional profile within the health or social care disciplines: physiotherapists, dietitians, nurses, dentists, physicians, pharmacists, and social workers were some of the represented professionals from the different disciplines. In terms of the educational representatives, deans and vice-deans of the different universities were also represented. From public administrations, representatives of city halls and public health agencies also attended. The profile of adolescents was difficult to include, being the group with less representation.

**Table 2:** Stakeholder consultation profile

Profile of the participants	Number of participants invited by each partner	Final number of participants
Professionals of the health or social disciplines such as psychologists, social workers, teachers, nurses, physiotherapists, dietitians, physicians, dentists, pharmacists, physical educators, sport scientists included in phases 1–5	10–12	45
People that ages Adolescent Young Adult (25–40) Adult (40–65) Elderly (> 65)	1 of each group (total of 4)	15
People that influence decisions on the content of educational programs: academic vice-rectors and vice-deans, professional associations, national platforms for the education of different professionals, etc.	6–8	9
Representatives of public administrations dealing with healthy ageing: health, social and welfare departments at different levels (city, region, country…)	2–3	10

Overall, there was a clear consensus on the relevance of the topic, as well as the need to reconsider the definition of healthy ageing and its impact by including a lifespan and participative approach on its meaning, design and implementation. Additionally, the necessity for an inter-/multiprofessional approach to expand the boundaries of current mainstream ideas about healthy ageing (mostly situated in older ages) was emphasised. The design of a professional curriculum focusing on general and specific competencies entailing biological, psychological, social, professional and cultural aspects and the conception of the person as a whole was also commented on among the different stakeholder meetings.

Furthermore, there was a general consensus on the necessity to redefine healthy ageing and starting to develop a culture that includes this concept from the beginning of life. This vision was not seen as just a responsibility for health, social and other professionals but of society as a whole. In the view of the different stakeholders, the integration of what is considered healthy ageing should be developed from the start of educational life, starting with undergraduate training with the possibility to complement it in postgraduate training.

With regard to the stakeholder views of the competencies identified in the literature, the general impression was of agreement with the proposed items, although missing areas were also identified. In the area of *Communication* with users, families, caregivers and the community, the German stakeholders’ meeting included the ability to convey a sense of security to users/families, the Spanish one added fostering relationships across generations and domains to promote socialisation, the Dutch group requested the inclusion of the ability to deal with resistance and group dynamics, while additions from the Kosovo stakeholder meeting included the ability to develop a person-centred approach to communication and user engagement. With regard to professional communication, the German and Kosovan stakeholders added the recognition of the need for collaboration and interprofessional networking to promote healthy ageing for each user. In the thematic area of technology, knowledge about tele-health was mentioned by the Kosovan stakeholders. Last, the transfer of evidence-based practice to the general population was included by the German and Spanish stakeholders and the development of governmental and non-governmental communication campaigns on healthy ageing by the Kosovan team.

In the *Knowledge* domain, the German stakeholders included having knowledge to promote the healthy ageing of children and the interaction within their, e.g. family/school/leisure time settings, knowledge of national and international jurisdictions and legal frameworks was added by the Dutch stakeholders, knowledge of developmental psychology, economics, social and health systems was proposed in the Kosovan stakeholder meeting and the Spanish stakeholders proposed the inclusion of knowledge on drug-nutrient interactions and training in supplements and products that society now consumes. In the thematic area of social and environmental aspects, including knowledge of norms and values of the client and the society shaped by culture, religion, spirituality, differences between generations and sexual diversity was requested by Germany and Spain. The German stakeholders also added knowledge of the role of social service, community recreation and health providers for older people and opportunities for voluntary work, knowledge of strategies and the development of concepts to overcome socioeconomic and educational barriers and foster the facilitation of personal resources (i.e. home visits/Clients environment/Community therapy, technological interventions and suitable tools to support older adults in their daily life). The Spanish stakeholders also asked for knowledge about sexuality and intimacy in the elderly to be included, as well as new family models, whereas the Finnish team incorporated knowledge on how to develop environments and processes to support healthy ageing in the long-term in Europe. Basic knowledge about developmental theories, healthy ageing and the importance of early childhood was also included by Finnish stakeholders, which constituted a new thematic area in the knowledge domain.

In the domain of *Technical Skills*, several countries' stakeholders indicated the inclusion of the ability to provide the integrated management of common conditions and complex health care needs faced by older populations and also conditions such as diabetes, medication dependence or abuse. The German stakeholders requested the incorporation of taking legal action if necessary when identifying neglect or abuse, as well as the ability to solve complex and unforeseen situations in the treatment of the elderly and to assess when therapy is indicated and when other caregivers should be involved. Both the Kosovan and Spanish stakeholders referred to the skill of implementing evidence-based solutions for the health and well-being of people and of integrating research into practice. The Finnish stakeholders included having self-care skills. Several additions were made to the Health Promotion Skills thematic by the German and Spanish stakeholders, including the promotion of healthy eating/food intakes habits, behavioural change and adherence promoting skills and soft skills.

Critical thinking was included in the *Clinical Reasoning* domain by the Spanish stakeholders. In the *Attitudes* domain, several items were included by the Kosovan and Dutch stakeholders, such as being bold and going against existing patterns and mindsets (if you really want to change things and change behaviour), adaptability, perseverance, looking at possibilities and not problems, equality in treatment, commitment, dedication, benevolence and impartiality with all people, seeking lifelong learning, respecting patient values and adopting “a friendly approach” and holistic understandings of healthy ageing.

## Discussion

Healthy ageing is one of the greatest global challenges for European (and other) societies and health and social professionals will have a key role in its promotion. To the best of our knowledge, this is the first study to analyse, from an international perspective, the competencies that have been described for health and social professionals to promote healthy ageing across the lifespan. As recommended in the development of scoping reviews, we followed an iterative procedure in order to maximise the relevance of the search, while preserving comprehensiveness. Decisions made along this process led to a final bibliographic sample of 20 documents (four studies retrieved from the scientific literature and 16 from the grey literature) and the participation of 79 international stakeholders from seven countries and a variety of profiles.

The definite inclusion of only 20 documents as a result of the literature search positioned the international consultation with the different stakeholders as a very valuable source of information for our research. Although not being a compulsory step when conducting a scoping review, it is highly recommended not to avoid this phase, as it helps with the inclusion and analysis of multifaceted perspectives and helps to identify gaps in the literature (Levac et al. 2010). Indeed, counting on 79 health and social professionals, researchers, policymakers and the general public of different ages (from adolescents to older citizens) from seven European countries increases the reach and variety of input that can be collected to enrich and complement the results of the literature search. Because the selection of stakeholders followed the same criteria in different countries, we not only captured the unique views of different health and social professions, different professional occupations (clinical practice, research, public health, etc.) and different demographic profiles, but also the sociocultural connotations of healthy ageing in contexts that are so similar yet so different, such as Finland, Germany, The Netherlands, Poland, Portugal, Kosovo and Spain. In this way, the representativeness and applicability of the results in the European region is enhanced. One more aspect to highlight is that our research team, formed of different professional profiles and healthcare providers, has also served as a platform for constant consultation which helped to enrich our analysis throughout the research process.

The scarcity of scientific and grey literature on the competences linked to healthy ageing needed by health and social care professionals clearly shows a shortage in research on the topic. The fact that we found only 4 records in the scientific literature and 16 in the grey probably reflects that despite the urgency of the matter, it has only recently received scholarly attention. Indeed, all of the identified studies in our literature search were published between 2014 and 2020, despite the time range for the inclusion of the studies being from 2000 to 2021.

In terms of the professional competencies to promote healthy ageing, we identified 142 competences referred to professionals from different disciplines in the health and social care field. Half of the identified documents described multidisciplinary competences and we only found documents focusing on profession-specific competencies for nurses, social workers, medical doctors and mental health professionals. Competences regarding other health and social professionals (physical therapists, speech therapists, dieticians, dentists, occupational therapists, physical educators, among others) were not found, although all of them play an important role in the promotion and management of healthy ageing. This indicates that, so far, more importance has been given to this topic by certain professions, highlighting a lack of interdisciplinary and person-centred approaches when considering healthy ageing which has been described as a major challenge by other authors (Young and Siegel 2016) and was also emphasised in the stakeholder consultations. As we draw on a few paragraphs later, an excessive focus on the older adult instead of on the whole lifespan, as well as a focus on treatment versus promotion/primary prevention, when describing such professional competences for healthy ageing may explain these results.

The competences identified depict a professional with a sound knowledge of the physiological and pathophysiological aspects of ageing and how the social, cultural and other environmental factors shape it and who is able to consider them when evaluating and providing evidence-based care to the user or communities who age. In this regard, the professional is especially sensitive to how these factors can create or amplify inequalities and is able to build steps to narrow or avoid them. Importantly, professionals orientated towards healthy ageing will work from the paradigm of complexity, applying critical thinking and enhancing inter/transdisciplinary attention and favouring the growth, autonomy and empowerment of the user (and the community). Therefore, professionals promoting health ageing y need to be able to work jointly with other disciplines. They also need to be able to adopt a person-centred communication style, by establishing and sustaining effective communication with the user, family, caregiver and relevant stakeholders and to use technology to facilitate this communication and care. The literature review findings also describe a professional who is aware of the existing policies and programs to promote healthy ageing and who is able to design, develop, implement and evaluate programs and activities themselves. In this sense, the professional promoting healthy ageing across the lifespan should be able to demonstrate leadership skills to generate actionsaligned with a vision of healthy ageing that involve individuals, communities and stakeholders to convey changes in the lifestyle and the structures that support them. Last, experts on healthy ageing will need to update their knowledge regularly, following the notion of lifelong learning. Such characteristics should contribute to more efficient healthy and social systems that are able to better preserve and promote the health of European citizens across generations.

In our research, half of the documents described competences referring explicitly to the attention to the older adult population, while only six framed the competences to promote healthy ageing as something to be applied throughout the life course. These results echo the general trend (i.e. the WHO’s World report on Ageing and Health (2015), or the Healthy Ageing Decade which define ageing from a life-course perspective but focuses only on the later stages of life) and constitute a call for action to draw attention to the whole lifespan when fostering healthy ageing. These results also point towards conceptual and practical uncertainties such as how the promotion of healthy ageing is differentiated from overall health promotion. In this review, we chose to only review documents that explicitly addressed healthy ageing to better grasp the current situation. We reflect on the conceptual differences and practical implication of choosing one or another term elsewhere (manuscript under preparation).

In any case, what becomes clear from the literature review part of this study is the fact that the workforce's training to promote healthy ageing across the lifespan is still in its infancy and its development will be key to deploying a more strategic response to the challenge of healthy ageing, which is currently under-addressed by most sectors and stakeholders.

In the different stakeholder meetings, a clear claim to start working on a cultural change for what we mean for healthy ageing from the very beginning of childhood education was shared, which also entails the design of a new perspective for the professionals that will be involved in the development of this new paradigm. Extended longevity is not synonymous with good health, especially if not tackled from the beginning of life (Scott 2021). Additionally, according to the different stakeholders consulted, the development of competencies to promote healthy ageing across the lifespan should not only be a responsibility of professionals, but of society as a whole, highlighting the importance of including a participatory approach in all of the decisions included on this matter and the need for a paradigm shift.

Our work presents several limitations. Conducting a scoping review involves following an iterative process including the constant reflection and debate of the research team about the elements embedded in the research development (objectives, search strategy, etc.) (Daudt et al. 2013). In our case, the decisions made to enhance the precision of our search such as referring only to competences instead of a combination of “knowledge”, “skills”, “capabilities” and “abilities, differentiating the concept of healthy ageing from health promotion” and not including studies reporting perceptions of competences, may have narrowed the reach of our results. However, doing so we ensured that all of the included results were relevant for the goal of this work. Even though most of the references included come from the grey literature, implying that they have not been subject to the same quality procedures as the scientific literature, it provides us with an honest reflection of how scholarship on workforce capacitation on healthy ageing is still scarce. A further limitation is the fact that despite selecting a wide number of representatives on the topic to participate in the stakeholder consultation, some profiles were scarcely represented, such as teenagers, which might be a limitation that should be improved in future research.

The comprehensive systematic search of electronic databases from the health, educational and social field, grey literature, in the different languages of the consortium partners and the consultation phase and a sound theoretical foundation are strengths of this review, which allow us to identify the state of the art on the topic and some recommendations for research.

The next steps in this line of work include the organisation of the identified competences into a structured framework (i.e. CANMeds model), the identification of educational levels at which the different competences should be learned and particularities amongst disciplines. Future research should investigate other potentially important professional competences and address how to best implement these and how to favour current social and healthcare systems integrate the view of healthy ageing across the lifespan*.*
